# A Case of Dual Septic Foci in Both the Joint and Pleural Cavity Caused by Streptococcus agalactiae

**DOI:** 10.7759/cureus.57160

**Published:** 2024-03-29

**Authors:** Fataou Saley Younoussa, Elmostafa Benaissa, Yassine Ben Lahlou, Mostapha Elouennass, Mariama Chadli

**Affiliations:** 1 Bacteriology Laboratory, Faculty of Medicine and Pharmacy, Mohammed V Military Training Hospital, Mohammed V University, Rabat, MAR

**Keywords:** group b streptococcus, adult, streptococcus agalactiae, septic arthritis, infectious pleurisy

## Abstract

Group B Streptococcus (GBS or *Streptococcus agalactiae*) is a common component of the human flora. However, infections in adults are infrequent, and occurrences of infectious pleurisy or septic arthritis are exceedingly uncommon. To our knowledge, the concurrent manifestation of both conditions has not been previously documented.

We present the case of a 61-year-old man who exhibited an unusual association of infectious pleurisy and septic arthritis in the knee, both attributed to GBS. The patient was admitted to the hospital due to thoracic pain and discomfort in the left knee. Clinical examination revealed a pleural effusion in the left lung and arthritis in the left knee. Synovial and pleural fluid samples were sent to the bacteriology laboratory for cytobacteriological examination, confirming the presence of GBS in both fluids.

The patient is diabetic and has a history of undergoing total cystoprostatectomy for a urothelial tumor, with the placement of a mono J catheter. The prevailing hypothesis suggests that the colonization of the mono J catheter, followed by hematogenous dissemination, is the probable source of the infection.

This unusual clinical case underscores GBS's ability to induce severe invasive infections in adults, particularly in those with underlying medical conditions.

## Introduction

*Streptococcus agalactiae*, commonly known as Lancefield Group B Streptococcus (GBS), is a typically harmless bacterium present in the genital tract of healthy women, as well as in the gastrointestinal and urinary tracts of healthy adults [1]. Although GBS infections are more frequently encountered in newborns and can manifest severely, causing conditions like bacteremia and meningitis [2], instances of this opportunistic pathogen causing bacteremia in elderly individuals or adults with compromised immune systems, such as those with diabetes or cancer, have been documented [2,3]. Nevertheless, its association with infectious pleurisy or septic arthritis is rare [4,5], with no reported cases of both conditions being attributed to GBS simultaneously.

A meta-analysis focusing on invasive GBS infections in nonpregnant adults revealed a low incidence of 2.86 cases per 100,000 inhabitants [6], with septic arthritis accounting for only 10.2% of cases [7].

We report a case involving a 61-year-old patient who developed an exceptional association between infectious pleurisy and septic arthritis of the knee, both attributable to *Streptococcus agalactiae*.

## Case presentation

We describe a case of a 61-year-old man, a chronic smoker who quit three years ago, with type 2 diabetes under treatment. He has a surgical history of total cystoprostatectomy 19 months ago for a urothelial tumor with placement of a mono J catheter. He was hospitalized due to thoracic side pain and discomfort in the left knee.

**Figure 1:**
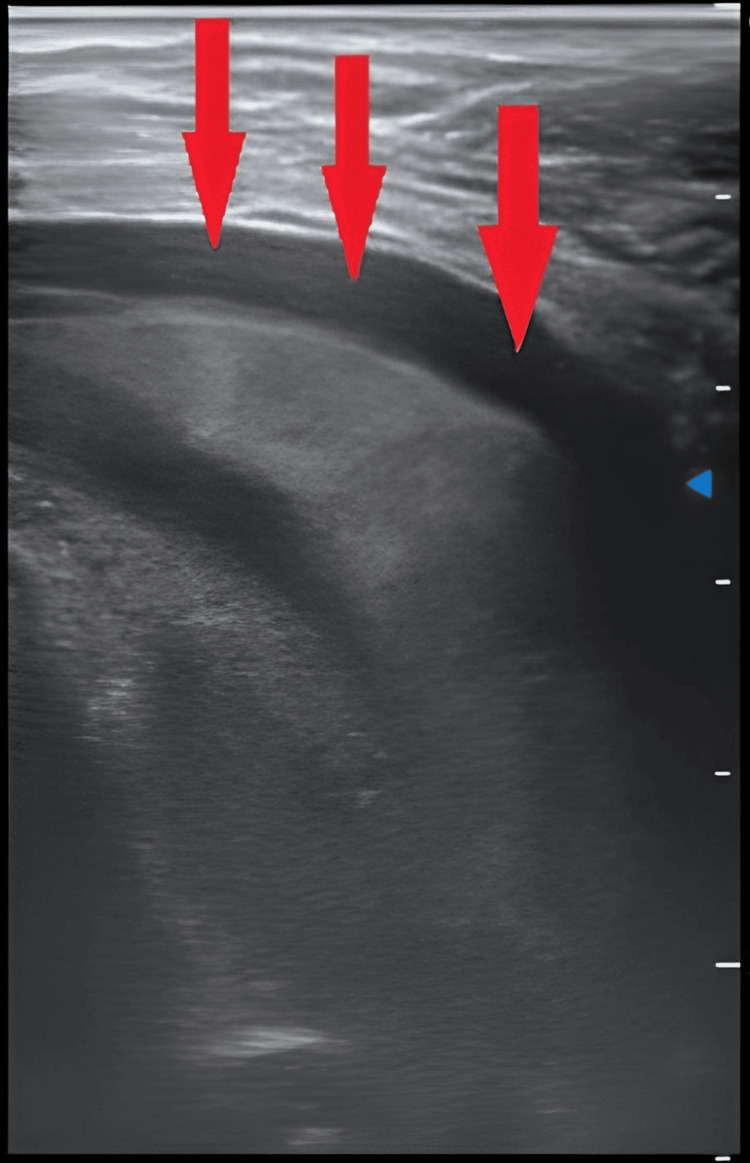
Chest X-ray showing a left pleural effusion

**Figure 2:**
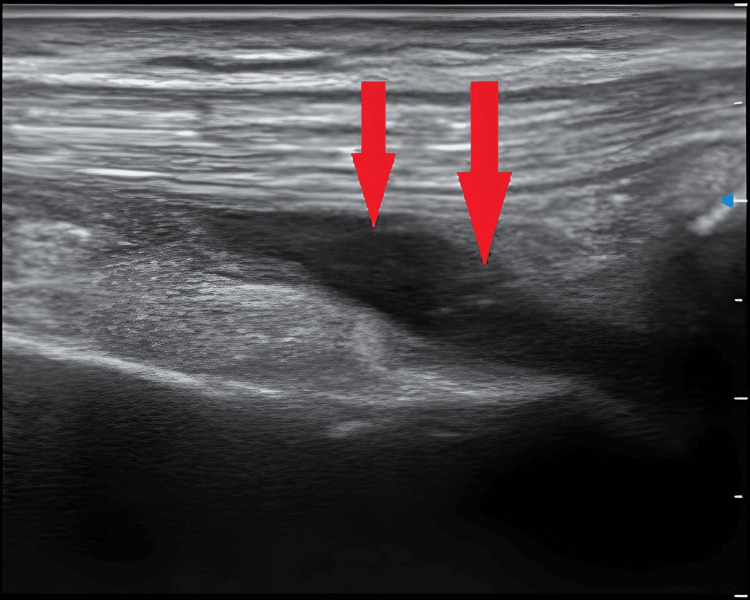
Ultrasound of the left knee demonstrating a collection in the quadricipital fossa

On clinical examination, the patient was eupneic. A left-sided pleural effusion and knee arthritis on the same side were found. Chest X-ray revealed a basithoracic opacity on the left with a watery appearance corresponding to a small amount of pleural effusion (Figure 1), while ultrasound of the left knee showed effusion with infiltration of the soft tissues, suggesting a collection in the quadricipital fossa (Figure 2).

**Figure 3:**
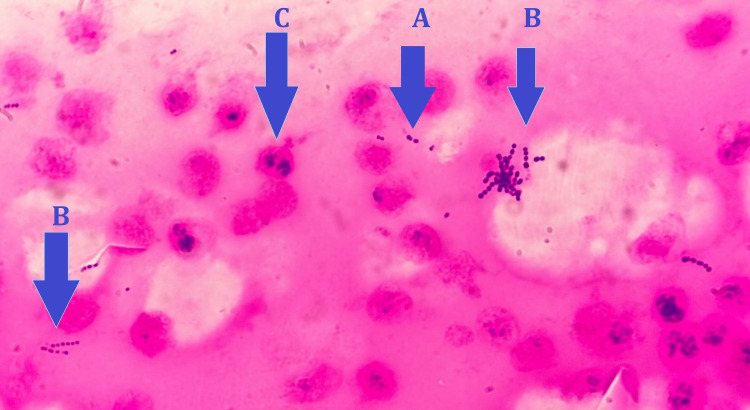
Gram-stained smear of the pleural fluid showing Gram-positive cocci in pairs (A) and chains (B) and neutrophilic polymorphonuclear cells(C)

**Figure 4:**
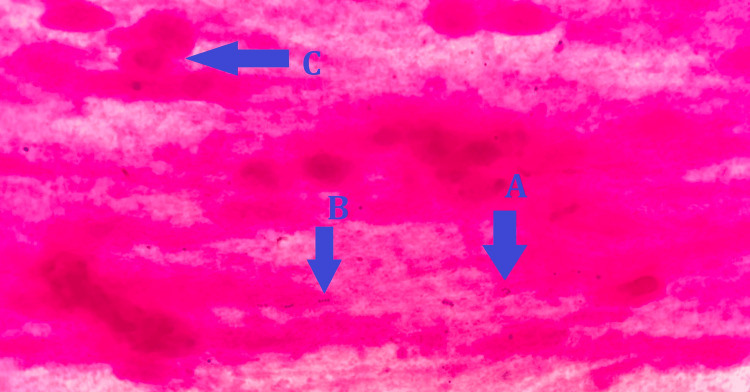
Gram-stained smear of the joint fluid showing Gram-positive cocci in pairs (A) and chains (B) and neutrophilic polymorphonuclear cells (C)

The synovial and pleural fluids were sent to the bacteriology laboratory for cytobacteriological examination. Cytological examinations using a Kova Slide® cell counter showed both to have a leukocyte count exceeding 10^6^ cells per milliliter. The leukocyte formulas revealed neutrophilic polymorphonuclear cell rates of 95% in the pleural fluid and 93% in the synovial fluid. Microcrystals were not found during direct examination of the latter. Gram staining smears demonstrated a rich bacterial flora consisting of numerous Gram-positive cocci in pairs and chains, both in the pleural fluid (Figure 3) and in the synovial fluid (Figure 4).

Both fluids were cultured on appropriate culture media (blood agar and enriched chocolate agar) and incubated in a CO2 incubator at 37°C. Additionally, an enrichment on brain-heart infusion broth was performed.

After 24 hours of incubation, both cultures returned positive with a monomorphic aspect of the colonies. Microscopic examination after Gram staining helped guide identification by allowing the selection of biochemical tests to be performed. The genus and species diagnosis of both cultures was established using API®20 Strep strips. The results indicated that the species is *Streptococcus agalactiae* for both fluids.

Antimicrobial susceptibility testing revealed an identical sensitivity profile for both strains of *Streptococcus agalactiae*. They were susceptible to all antibiotics tested (penicillin G, norfloxacin, levofloxacin, moxifloxacin, gentamicin, vancomycin, teicoplanin, erythromycin, tetracycline, linezolid, and rifampicin).

Concurrent blood cultures were negative. Therefore, the cytobacteriological examination of the pleural fluid and synovial fluid allowed for the diagnosis of pleurisy and septic arthritis caused by the same pathogen: *Streptococcus agalactiae*.

The patient was treated with third-generation cephalosporin combined with gentamicin (for three days) and moxifloxacin for a duration of four weeks, resulting in good clinical, biological, and radiological improvement.

## Discussion

*Streptococcus agalactiae* is an encapsulated Gram-positive coccus. Its polysaccharide capsule is characterized by the Lancefield group B antigen; it is one of the main virulence factors associated with the bacterium's invasive capacity [8]. Infections with GBS are more common in pregnant women and newborns [2]. GBS is part of the normal flora in healthy adults and acts as an opportunistic pathogen, exceptionally transitioning from asymptomatic carriage to noninvasive or invasive disease. In certain conditions, it can cause infectious pleurisy or septic arthritis. However, to our knowledge, the association of both conditions has not yet been described.

The results of the largest and most recent meta-analysis conducted between 1975 and 2018 on the epidemiology of invasive GBS infections in nonpregnant adults revealed an incidence of 2.86 cases of invasive infections per 100,000 population [6].

Knowledge of the microbiological landscape of infectious pleurisy remains incomplete [9]. The most recent reported case of infectious pleurisy due to *Streptococcus agalactiae *dates back to 2002 [10].

Numerous studies have analyzed the microorganisms responsible for infectious pleurisy, revealing that the prevalence of *Streptococcus agalactiae* is very low, ranging between 0% and 3.9% across different series. It's noteworthy that this latter series includes newborns, postpartum, and nonpregnant adults [9,10].

Likewise, the involvement of GBS in septic arthritis is uncommon. Thorough retrospective analyses have consistently shown that GBS accounts for merely 5% to 10% of septic arthritis instances, with the knee being the predominant site, mirroring the situation observed in our patient [4,7].

Cohort studies find that diabetic and obese patients have a fourfold higher risk of invasive GBS infection [7,11]. Other underlying conditions have also been mentioned, such as cancer, chronic skin diseases, heart failure, cardiovascular diseases, chronic kidney disease, and neurological disorders, which were present in more than 15% of patients [7].

Therefore, our patient exhibits at least one of these risk factors, as he is diabetic and underwent total cystoprostatectomy secondary to a urothelial tumor.

In invasive GBS infections, blood cultures are not systematically positive, as demonstrated by the case of our patient, whose blood cultures performed simultaneously with the joint fluid culture were negative. For septic arthritis, for example, blood cultures are positive in approximately one out of four cases [12]. Isolates of GBS are generally sensitive to beta-lactams [13], as is the case of our patient.

Microbiological analysis of samples from both pleural and joint fluids confirmed the presence of *Streptococcus agalactiae*, thus confirming the simultaneous occurrence of infectious pleurisy and septic arthritis.

The incidence of invasive GBS infections increased with the use of invasive medical devices such as urinary or intravenous catheters [6]. In our patient's case, colonization of the mono J catheter placed since the cystoprostatectomy followed by hematogenous dissemination is believed to be the origin of his infection. This observation thus contributes to enriching the literature data regarding *Streptococcus agalactiae*'s ability to induce severe invasive infections in adults.

## Conclusions

Invasive GBS infection is uncommon in adults, especially concurrent involvement of the pleural cavity and joints. Risk factors such as diabetes and cancer are frequently associated. A genitourinary or gastrointestinal entry point cannot be ruled out.
